# Statin and aspirin as adjuvant therapy in hospitalised patients with SARS-CoV-2 infection: a randomised clinical trial (RESIST trial)

**DOI:** 10.1186/s12879-022-07570-5

**Published:** 2022-07-09

**Authors:** Nirmal Ghati, Sushma Bhatnagar, Manjit Mahendran, Abhishek Thakur, Kshitij Prasad, Devesh Kumar, Tanima Dwivedi, Kalaivani Mani, Pawan Tiwari, Ritu Gupta, Anant Mohan, Anita Saxena, Randeep Guleria, Siddharthan Deepti

**Affiliations:** 1 Department of Cardiology, Jai Prakash Narayan Apex Trauma Center, All India Institute of Medical Sciences (AIIMS), New Delhi, India; 2 Department of Onco-Anaesthesia, Dr. B.R.A Institute-Rotary Cancer Hospital, All India Institute of Medical Sciences (AIIMS), New Delhi, India; 3 Department of Cardiology, All India Institute of Medical Sciences (AIIMS), New Delhi, India; 4 Department of Laboratory Medicine, National Cancer Institute (Jhajjar, Haryana), All India Institute of Medical Sciences (AIIMS), New Delhi, India; 5 Department of Biostatistics, All India Institute of Medical Sciences (AIIMS), New Delhi, India; 6 Department of Pulmonary Medicine and Sleep Disorders, All India Institute of Medical Sciences (AIIMS), New Delhi, India; 7 Department of Laboratory Oncology, Dr. B.R.A Institute-Rotary Cancer Hospital, All India Institute of Medical Sciences (AIIMS), New Delhi, India; 8 Department of Cardiology, Cardiothoracic Sciences Centre, All India Institute of Medical Sciences, Ansari Nagar, New Delhi, 110029 India

**Keywords:** COVID-19, Statin, Aspirin, WHO ordinal scale, Serum IL-6

## Abstract

**Background:**

Statins and aspirin have been proposed for treatment of COVID-19 because of their anti-inflammatory and anti-thrombotic properties. Several observational studies have shown favourable results. There is a need for a randomised controlled trial.

**Methods:**

In this single-center, open-label, randomised controlled trial, 900 RT-PCR positive COVID-19 patients requiring hospitalisation, were randomly assigned to receive either atorvastatin 40 mg (Group A, n = 224), aspirin 75 mg (Group B, n = 225), or both (Group C, n = 225) in addition to standard of care for 10 days or until discharge whichever was earlier or only standard of care (Group D, n = 226). The primary outcome variable was clinical deterioration to WHO Ordinal Scale for Clinical Improvement ≥ 6. The secondary outcome was change in serum C-reactive protein, interleukin-6, and troponin I.

**Results:**

The primary outcome occurred in 25 (2.8%) patients: 7 (3.2%) in Group A, 3 (1.4%) in Group B, 8 (3.6%) in Group C, and 7 (3.2%) in Group D. There was no difference in primary outcome across the study groups (P = 0.463). Comparison of all patients who received atorvastatin or aspirin with the control group (Group D) also did not show any benefit [Atorvastatin: HR 1.0 (95% CI 0.41–2.46) P = 0.99; Aspirin: HR 0.7 (95% CI 0.27–1.81) P = 0.46]. The secondary outcomes revealed lower serum interleukin-6 levels among patients in Groups B and C. There was no excess of adverse events.

**Conclusions:**

Among patients admitted with mild to moderate COVID-19 infection, additional treatment with aspirin, atorvastatin, or a combination of the two does not prevent clinical deterioration.

*Trial Registry Number* CTRI/2020/07/026791 (http://ctri.nic.in; registered on 25/07/2020)

**Supplementary Information:**

The online version contains supplementary material available at 10.1186/s12879-022-07570-5.

## Introduction

Statins have been proposed for the treatment of COVID-19 based on their pleiotropic anti-inflammatory, antioxidant, and immunomodulatory properties [1]. The rationale stems from several observational and randomised studies in bacterial and viral pneumonias [2, 3] and severe sepsis [4, 5] that have reported a significant association between prior statin use and reduction in duration of hospital stay, intensive care unit (ICU) admissions, and mortality. In the past two years, observational reports of prior or current use of statins in COVID-19 have been published with mixed results [6–10].

Similarly, aspirin has been proposed as a potential repurposed drug for COVID-19. The antiplatelet agent has been shown to have anti-inflammatory and antiviral actions, even at low doses [11–13]. The antithrombotic properties may be beneficial in the hypercoagulable state which is a key feature of severe COVID-19 infection [14]. Several observational studies have shown decreased mortality in severe COVID-19 infection with pre-hospitalisation use of aspirin [15–17]. However, a recent randomised trial by the RECOVERY collaborative group has reported that the use of aspirin was not associated with reductions in mortality or the risk of progression to invasive mechanical ventilation or death in patients hospitalised with COVID-19 [18].

Both drugs are affordable and globally available. With this background, we conducted a randomised controlled trial to evaluate the benefits and safety of adding statins and aspirin separately or together to the standard of care in patients hospitalised with mild to moderate COVID-19 infection.

## Methods

### Study design

The details of the trial design have been published [19]. Briefly, the study was a single-centre, prospective, four-arm parallel design, open-label randomised controlled trial. It was conducted at a designated COVID-19 facility established at the National Cancer Institute (NCI), Jhajjar, Haryana, an outreach centre of All India Institute of Medical Sciences (AIIMS), New Delhi. The trial was approved by the institutional ethical committee and was conducted in accordance with the 1964 Helsinki Declaration and its later amendments. Recruitment of patients started on 28th July 2020, and enrolment was completed on 27th January 2021. Written informed consent was obtained from all participants or their legal representatives. The trial was registered in the Clinical Trials Registry of India [http://ctri.nic.in; Registration Number: CTRI/2020/07/026791 (registered on 25/07/2020)].

### Participant recruitment and baseline assessment

All RT-PCR positive COVID-19 patients, ≥ 40 years and < 75 years of age, requiring hospitalisation due to symptoms [World Health Organization (WHO) Ordinal Scale for Clinical Improvement 3 to 5 (Table 1)] were included in the trial. Patients with a critical illness (WHO Ordinal Scale for Clinical Improvement > 5), documented significant liver disease/dysfunction [aspartate transaminase (AST)/alanine aminotransferase (ALT) > 240 IU/L], myopathy, and rhabdomyolysis [creatine phosphokinase (CPK) > 5 × normal], known allergy or intolerance to statins or aspirin, prior statin or aspirin use in the last 30 days, history of active gastrointestinal bleeding in the past three months, coagulopathy, thrombocytopenia (platelet count < 100,000/dL), pregnancy, active breastfeeding, or inability to take oral or nasogastric drugs were excluded. Patients refusing consent and taking drugs known to have significant interaction with atorvastatin [including cyclosporine, protease inhibitors, fibrates, niacin, azole antifungals (itraconazole, ketoconazole), clarithromycin, and colchicine] were excluded from the trial. Patients on parenteral anticoagulation for COVID-19 infection were not excluded [19].

**Table 1:** WHO ordinal scale for clinical improvement

Patient State	Descriptor	Scale
Uninfected	No clinical and virological evidence of infection	1
Ambulatory	No limitation of activities	2
	Limitation of activities	3
Hospitalised mild disease	Hospitalised, no oxygen therapy	4
	Oxygen by mask or nasal prong	5
Hospitalised severe disease	Non-invasive ventilation or high-flow oxygen	6
	Intubation and mechanical ventilation	7
	Ventilation + additional organ support -pressors, ECMO, RRT	8
Dead	Death	9

*ECMO* extracorporeal membrane oxygenation, *RRT* renal replacement therapy, *WHO* World Health Organisation

Demographic information, including age, gender, and residential address was recorded for all recruited patients. All patients were clinically evaluated for comorbid conditions including diabetes mellitus, hypertension, coronary artery disease, heart failure, ischemic stroke, chronic kidney disease and chronic liver disease, drug history, and COVID-19 related symptoms and signs.

### Randomisation and intervention

The study used a four-arm parallel-group design. A computer-generated permuted block randomisation with mixed block size was used to randomise the participants in a 1:1:1:1 ratio into four groups: Group A (atorvastatin), Group B (aspirin), Group C (combination of aspirin and atorvastatin), and Group D (control). All groups received conventional medical therapy according to the institute’s COVID-19 treatment protocol (Additional file 1: eFigure 1) and the treating physician’s clinical judgment. Atorvastatin and aspirin were prescribed at doses of 40 mg and 75 mg, respectively once daily for 10 days or until discharge, whichever was earlier. The doses and duration of therapy (10 days) were decided based on studies on the use of statins and aspirin in non-COVID pneumonias.

### Follow up

All study participants were followed up for ten days or until hospital discharge whichever was later. Patients with early discharge (due to clinical improvement and patient’s preference for home isolation) were followed up by alternate day telephonic contact till the 10th day of the drug regimen. Serum creatine phosphokinase (CPK), liver function test (LFT), troponin I (Trop-I), and serum inflammatory biomarkers including C-reactive protein (CRP), and interleukin 6 (IL-6) were repeated on 5th day of study enrolment or 7th day after symptom onset, whichever was later. Decisions regarding other drugs and investigations were based on the institute’s COVID-19 management protocol (Additional file 1: eFigure 1) and the treating physician’s clinical judgement. There were no major changes in conventional therapy protocol during the study duration.

### Outcome variables

The primary outcome variable was clinical deterioration to WHO Ordinal Scale ≥ 6 [i.e., requirement for high flow oxygen, non-invasive ventilation, endotracheal intubation, administration of vasopressor agents, renal replacement therapy, extra corporeal membrane oxygenator (ECMO) requirement, and mortality] [19]. The secondary outcomes were change in serum inflammatory markers (CRP and IL-6), and troponin I level from baseline (time zero) to 5th day of study enrolment or 7th day after symptom onset, whichever was later [19]. Other clinical outcomes that were assessed included clinical deterioration (≥ 1 increase in baseline WHO Ordinal Scale), progression to shock, requirement of mechanical ventilation, length of hospital stay, and in-hospital mortality [19]. Adverse drug effects like myalgia [severe muscle pain or aches [CPK < Upper limit of normal (ULN)], myopathy (unexplained muscle pain or weakness accompanied by CPK > 10 × ULN), rhabdomyolysis (severe myopathy with CPK > 40 × ULN and myoglobinuria with or without acute renal failure), hepatotoxicity (ALT or AST > 3 × ULN), minor bleeding (BARC bleeding type 1 and 2 i.e. bleeding that is not actionable and does not cause the patient to seek treatment, bleeding requiring a healthcare assessment or less invasive treatment such as heavy menstrual bleeding, ecchymosis, or epistaxis), and major bleeding [BARC bleeding type ≥ 3 i.e., significant blood loss requiring blood transfusion, bleeding into a critical closed space (e.g., intracranial bleeding, compartment syndrome), bleeding requiring an intervention for management (e.g., surgery, interventional radiology procedures, endoscopic treatments), and fatal bleeding] were also examined in the trial to assess the safety of the interventions [19].

### Sample size calculation

As there were no previous studies that had evaluated the role of statins and aspirin in COVID-19, formal sample size calculation was not feasible. It was decided to determine the final sample size based on the results of the first 800 patients included in the study (200 in each arm). After the recruitment of 900 patients, the study was not extended further due to the results and also the closure of the facility due to the dwindling number of COVID-19 cases in Delhi, India in January 2021.

### Statistical analysis

The quantitative variables were summarised through descriptive statistics, i.e., median (p25-p75), and the categorical variables were summarised through frequency (n) and percentage (%). Modified Intention-to-treat [(modified ITT), after excluding inadvertently randomised ineligible patients], true ITT, per-protocol (PP), and as per treatment received analyses were carried out for outcomes [19]. The primary outcome was compared between the groups using Chi-square test [19]. Serum biomarkers (CRP, IL-6, and Trop I) were tested for normality assumption using Shapiro-wilks test [19]. Variables that did not follow normal distribution were analysed using Wilcoxon rank-sum test and Wilcoxon signed-rank test. Other outcomes (time-to-event) were compared using Kaplan–Meier curve and log-rank test. The Cox proportional hazards model was used to calculate the hazards ratio and 95% Confidence Interval (CI). Safety outcomes were compared between the groups using Chi-square or Fisher’s exact test [19]. Two-sided P value < 0.05 was considered significant. The data were analysed using Stata 15.0 (*Stata Corp, 4905 Lakeway, Dr College Station, TX 77845 USA)* statistical software [19].

## Results

### Patient population

**Fig. 1:**
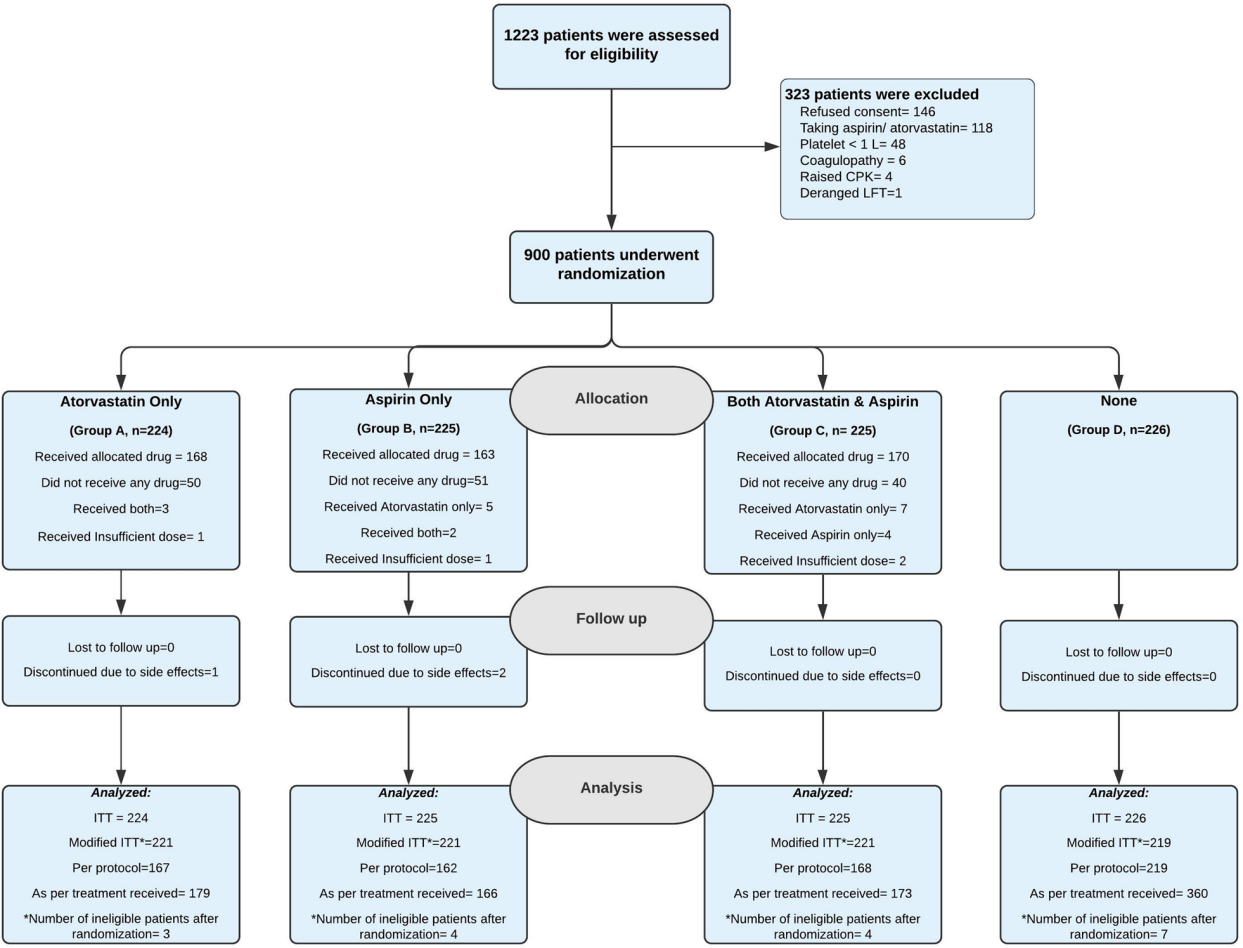
Consort flow diagram showing screening, recruitment, and randomisation of study participants. *CPK* Creatine phosphokinase, *LFT* Liver function test, *ITT* intention to treat

A total of 1223 COVID-19 patients admitted at the study centre between 28th July 2020 and 27th January 2021 were screened for eligibility. Among them, 900 patients were recruited and randomised into four study groups. Of these 900 participants, 224 patients were allocated to Group A, 225 to Group B, 225 to Group C, and 226 to Group D (Fig. 1). Of these, 3 patients in Group A, 4 patients in Group B, 4 patients in Group C, and 7 patients in group D were found to be ineligible after randomisation (10 patients were taking aspirin and/or atorvastatin before randomisation, 4 patients had thrombocytopenia, 2 patients had coagulopathy, 1 patient had raised baseline CPK, and 1 patient had raised baseline liver enzymes). This information was not available at the time of recruitment but revealed subsequently and thus, these patients were excluded from the final analyses (modified ITT analysis) of the study. One patient in Group A and 2 patients in Group B discontinued the study drugs due to side effects. None of the patients were lost to follow-up.

The baseline characteristics of the study participants were similar across the study groups and are reported in Table 2. The median age of the participants was 52 [interquartile range (IQR) 46–59] years, and 650 (73.7%) patients were male. Of the 900 study participants, 724 (82.1%), 133 (15.1%) and 25 (2.8%) patients had baseline WHO Ordinal Scale of 3, 4, and 5 respectively. The baseline D-dimer level was 153 ng/ml (IQR 84–271 ng/ml) and there was no inter-group difference. The median levels of serum CRP, IL-6, and troponin I at baseline were 1.4 (IQR 0.3–5.8) mg/dl, 6 (IQR 1.8–22) pg/mL, and 0.005 (IQR 0.001–0.008) ng/mL, respectively. At the time of randomisation, 247 (28%) and 241 (27%) patients were receiving low molecular weight heparin (LMWH) and steroids respectively. The patients were randomised to the study intervention after a median period of 6 (IQR 4–8) days after the onset of symptoms.

**Table 2:** Baseline characteristics of study population

Baseline variables	Total population^#^ (n = 882)	Group A (Atorvastatin) (n = 221)	Group B (Aspirin) (n = 221)	Group C (Both) (n = 221)	Group D (None) (n = 219)
Age (years)	52 (46–59)	51 (45–59)	53 (46–60)	52 (46–57)	52 (46–60)
Sex–Male	650 (74)	166 (75)	160 (72)	163 (74)	161 (74)
Number of days since symptom onset	6 (4–8)	6 (3–8)	6 (4–8)	6 (4–9)	5 (4–8)
Comorbidities					
Diabetes	244 (28)	65 (29)	57 (26)	69 (31)	53 (24)
Hypertension	252 (29)	66 (30)	65 (29)	62 (28)	59 (27)
Coronary artery disease	10 (1)	2 (1)	2 (1)	4 (2)	2 (1)
Liver dysfunction	5 (1)	2 (1)	2 (1)	1 (0)	0 (0)
Chronic kidney disease	21 (2)	5 (2)	5 (2)	4 (2)	7 (3)
Symptoms					
Fever	572 (65)	149 (67)	136 (62)	147 (67)	140 (64)
Cough	496 (56)	122 (55)	119 (54)	127 (58)	128 (58)
Shortness of breath	256 (29)	58 (26)	66 (30)	76 (34)	56 (26)
Desaturation	82 (9)	16 (7)	18 (8)	28 (13)	20 (9)
Body ache	94 (11)	29 (13)	22 (10)	21 (10)	22 (10)
G I symptoms	85 (10)	20 (9)	24 (11)	21 (10)	20 (9)
Loss of smell	31 (4)	7 (3)	7 (3)	6 (3)	11 (5)
Loss of taste	49 (6)	11 (5)	12 (6)	13 (6)	13 (6)
Treatment received					
Hydroxychloroquine	87 (10)	29 (13)	18 (8)	19 (9)	21 (10)
Azithromycin	103 (12)	31 (14)	23 (10)	26 (12)	23 (11)
Remdesivir	182 (21)	39 (18)	51 (23)	51 (23)	41 (19)
Favipiravir	38 (4)	11 (5)	10 (5)	7 (3)	10 (5)
Doxycycline	177 (20)	48 (22)	48 (22)	36 (16)	45 (21)
Anticoagulation	247 (28)	66 (30)	64 (29)	63 (29)	54 (25)
Plasma therapy	2 (0)	0 (0)	0 (0)	1 (0)	1 (1)
Tocilizumab	5 (1)	1 (0)	1 (0)	2 (1)	1 (1)
Steroid	241 (27)	58 (26)	65 (29)	58 (26)	60 (27)
Baseline ordinal scale					
3	724 (82)	181 (82)	182 (82)	178 (81)	183 (84)
4	133 (15)	33 (15)	33 (15)	37 (17)	30 (14)
5	25 (3)	7 (3)	6 (3)	6 (3)	6 (3)
Blood investigations					
Hemoglobin (g/dL)	13 (11.7–14.3)	13.1 (11.8–14.3)	12.9 (11.7–14.4)	13.1 (12–14.3)	12.9 (11.6–14.2)
TLC (/μL)	5905 (4540 -7 710)	5670 (4530–7510)	5920 (4600–7670)	5890 (4480–7860)	6020 (4600–8000)
Platelet (× 10^3^ /μL)	200 (149–258)	203 (156–262)	210 (151–261)	196 (149–254)	187 (141–262)
Urea (mg/dL)	27 (21.4–36.4)	25.7 (21–36.4)	25.7 (21.4–36)	27.00 (21–36.4)	28.50 (22–38.5)
Creatinine (mg/dL)	0.8 (0.7–0.9)	0.8 (0.7–0.9)	0.8 (0.7–0.9)	0.8 (0.7–0.9)	0.8 (0.7–0.9)
Total Bilirubin (mg/dL)	0.5 (0.4–0.7)	0.5 (0.4–0.7)	0.5 (0.4–0.7)	0.5 (0.4–0.7)	0.5 (0.4–0.8)
SGOT (IU/L)	38 (28.4–55)	37 (27.7–53)	41 (29–56)	38 (29–54.2)	36 (29–54)
SGPT (IU/L)	39 (25.5–60)	38 (24–57.2)	39.9 (27–61)	39 (26.5–59)	39 (25–60)
ALP (IU)	83 (67–108)	80 (65–110)	83 (20–109)	85 (67–108)	85 (69–104)
Ferritin (ng/mL)	220.6 (106–506)	202 (97.9–436)	249.85 (121–493.9)	225.5 (109–538.4)	220.5 (105.9–571.3)
D-Dimer (ng/mL)	153 (84–271)	138 (84–244)	159 (83–256)	152 (87–299)	168 (85–284)
PT (sec)	12.2 (11.5–13)	12.3 (11.5–13.1)	12.3 (11.6–13.2)	12.1 (11.45–12.9)	12.1 (11.5–13)
INR	1.1 (1–1.1)	1.1 (1–1.1)	1.1 (1–1.1)	1.1 (1–1.11)	1 (1–1.1)
CRP (mg/dl)	1.4 (0.3–5.8)	1.4 (0.3–5.3)	1.5 (0.3–7)	1.2 (0.3–6.3)	1.2 (0.3–4.9)
IL-6 (pg/mL)	6 (1.8–22)	6.8 (2–25.4)	5.5 (1.8–22)	7.9 (2–28.6)	5.2 (1.4–14.7)
Trop I (ng/mL)	0.005 (0.001–0.008)	0.006 (0.001–0.007)	0.004 (0.001–0.008)	0.005 (0.001–0.008)	0.005 (0.001–0.008)
CPK (IU/L)	96 (57–170)	92.50 (56–190)	98 (53–176)	97 (62–154)	97.50 (54–162)

Values are median (p25–p75) or n (%)

Local lab ranges: CRP ≤ 0.06 mg/dl, IL6 = 0–4.4 pg/ml, Ferritin = 22–322 ng/ml, D-dimer ≤ 500 ng/ml, CPK = 32–294 IU/L and Troponin I ≤ 0.04 ng/ml

SD = standard deviation, GI = gastrointestinal, TLC = total leucocyte count, SGOT = serum glutamic-oxaloacetic transaminase, SGPT = serum glutamic pyruvic transaminase, ALP = alkaline phosphatase, PT = prothrombin time, INR = international normalized ratio, CPK = creatine phosphokinase, CRP = C-reactive protein, IL-6 = interleukin 6, Trop I = troponin I, IU = International Unit, L = litre, mL = millilitre, mg = milligram, pg = picogram, ng = nanogram

^#^Modified intension-to-treat analysis

### Primary outcome

**Fig. 2:**
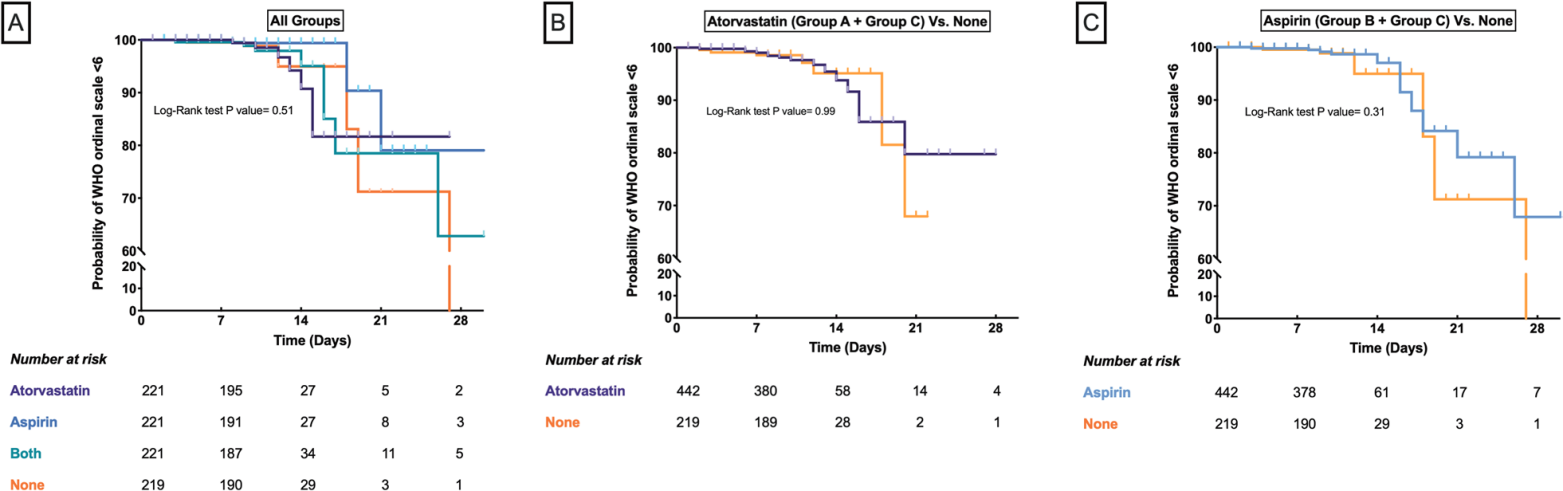
Probability of having WHO Ordinal Scale < 6 in the study groups over time. **A** is showing Kaplan–Meier estimates of freedom from primary outcome after initiation of the study drugs (atorvastatin, aspirin, and both) in comparison to the standard of care (modified ITT analysis). **B and C** are showing Kaplan–Meier estimates of probability of freedom from primary outcome in combined atorvastatin (Group A and Group C) and combined aspirin (Group B and Group C) groups respectively in comparison to the standard of care (modified ITT analysis). *CI* confidence interval, *HR* hazard ratio, *WHO* World Health Organisation

The primary outcome was observed in 25 (2.8%) patients including 7 (3.2%) patients in Group A, 3 (1.4%) in Group B, 8 (3.6%) in Group C, and 7 (3.2%) in Group D. Modified ITT analysis showed no difference in primary outcome across the study groups (P = 0.46) (Table 3, Fig. 2). A comparison of all study participants who received atorvastatin (combined Group A and Group C, n = 442) or aspirin (combined Group B and Group C, n = 442) with the control group (Group D, n = 219) also did not reveal any benefit of either drug in reducing the primary outcome [atorvastatin: HR 1.0 (95% CI 0.41–2.46), P = 0.99; aspirin: HR 0.7 (95% CI 0.27–1.81), P = 0.46] (Fig. 2). True ITT (900 patients), per-protocol (716 patients), and as-treated (878 patients) analysis were also carried out, but they did not materially affect the result (Table 3, Additional file 1: eFigures 2–4).

**Table 3:** Distribution of clinical outcomes in the study groups

Outcome variables		Group A (Atorvastatin)	Group B (Aspirin)	Group C (Both)	Group D (None)	P Value
Primary outcome (Progression to WHO ordinal score ≥ 6)						
Modified ITT (N = 882)	n/N (%)	7/221 (3.2)	3/221 (1.4)	8/221 (3.6)	7/219 (3.2)	0.46 ^a^
	HR (95% CI), P	0.98 (0.34–2.79), 0.97	0.40 (0.10–1.54), 0.18	1.00 (0.36–2.77), 0.99	1	
True ITT (N = 900)	n/N (%)	7/224 (3.1)	3/225 (1.3)	8/225 (3.6)	7/226 (3.1)	0.45 ^a^
	HR (95% CI), P	0.86 (0.30–2.47), 0.78	0.37 (0.09–1.43), 0.15	0.81 (0.28–2.29), 0.68	1	
Per protocol (N = 716)	n/N (%)	3/167 (1.8)	3/162 (1.8)	6/168 (3.6)	7/219 (3.2)	0.67^a^
	HR (95% CI), P	0.56 (0.14–2.16), 0.40	0.50 (0.13–1.96), 0.32	1.06 (0.36–3.16), 0.92	1	
As per treatment received(N = 878)	n/N (%)	3/179 (1.7)	3/166 (1.8)	6/173 (3.5)	13/360 (3.6)	0.52^a^
	HR (95% CI), P	0.45 (0.13–1.57), 0.21	0.46 (0.13–1.62), 0.23	0.95 (0.36–2.47), 0.89	1	
Other clinical outcomes						
Death						
Modified ITT		7/221 (3.2)	3/221 (1.4)	8/221 (3.6)	7/219 (3.2)	0.46^a^
True ITT		7/224 (3.1)	3/225 (1.3)	8/225 (3.6)	7/226 (3.1)	0.46 ^a^
Per protocol		3/167 (1.8)	3/162 (1.8)	6/168 (3.6)	7/219 (3.2)	0.67^a^
As per treatment received		3/179 (1.7)	3/166 (1.8)	6/173 (3.5)	13/360 (3.6)	0.52^a^
Mechanical ventilation						
Modified ITT		7/221 (3.1)	3/221 (1.3)	8/221 (3.6)	6/219 (2.7)	0.48^a^
True ITT		7/224 (3.1)	3/225 (1.3)	8/225 (3.6)	6/226 (2.7)	0.47 ^a^
Per protocol		3/167 (1.8)	3/162 (1.8)	6/168 (3.6)	6/219 (2.7)	0.72^a^
As per treatment received		3/179 (1.7)	3/166 (1.8)	6/173 (3.5)	12/360 (3.3)	0.59^a^
Shock						
Modified ITT		5/221 (2.2)	1/221 (0.4)	6/221 (2.7)	6/219 (2.7)	0.19^a^
True ITT		5/224 (2.2)	1/225 (0.4)	6/225 (2.7)	6/226 (2.7)	0.20 ^a^
Per protocol		2/167 (1.2)	1/162 (0.6)	5/168 (3)	6/219 (2.7)	0.32^a^
As per treatment received		2/179 (1.1)	1/166 (0.6)	5/173 (2.9)	10/360 (2.8)	0.27^a^
Clinical deterioration^b^						
Modified ITT		27/221 (12.2)	26/221 (11.7)	20/221 (9.0)	22/219 (10.0)	0.68
True ITT		27/224 (12.1)	26/225 (11.6)	21/225 (9.3)	22/226 (9.7)	0.74
Per protocol		17/167 (10.2)	14/162 (8.6)	15/168 (8.9)	22/219 (10)	0.95
As per treatment received		19/179 (10.6)	14/166 (8.4)	15/173 (8.7)	46/360 (12.8)	0.35
Hospital admission duration (days)^γ^						
Modified ITT		9 (8–11)	9 (8–11)	9 (8–12)	9 (8–11)	0.85
True ITT		9 (8–12)	9 (8–11)	9 (8–12)	9 (7–11)	0.65
Per protocol		9 (8–11)	9 (8–11)	9 (8–11)	9 (8–11)	0.69
As per treatment received		9 (8–11)	9 (8–11)	9 (8–11)	9 (7–12)	0.54

ITT intension-to-treat, WHO World Health Organisation

^γ^Median (p25-p75)

^a^Fisher exact test

^b^≥ 1 increase in baseline WHO clinical improvement ordinal score

### Secondary outcome

A ‘per-protocol’ and ‘as-treated’ analyses were done to assess any change in serum levels of biomarkers of inflammation and myocardial injury with study interventions. Among the three biomarkers, serum Trop I (P = 0.55) and CRP (P = 0.89) levels did not show any change with study interventions (Table 4). However, there was a significant decrease in serum IL-6 levels in Groups B and C. The reduction in serum IL-6 levels was greatest in the combined aspirin and atorvastatin group [Group C vs. Group D: median percentage (%) change − 53.56 vs. 0, P < 0.001], followed by aspirin only group (Group B vs. Group D: median % change − 40.42 vs. 0, P = 0.04). The change in IL-6 level was similar in the atorvastatin group and conventional group (Group A vs. Group D: median % change − 27.83 vs. 0, P = 0.10).

**Table 4:** Changes in biomarker levels in the study groups

Variables	Group A (Atorvastatin)	Group B (Aspirin)	Group C (Both)	Group D (None)	P Value
**CRP (mg/dl)**					
Pre	1.05 (0.25–3.65)	1.36 (0.20–6.72)	0.95 (0.18–4.74)	1.05 (0.23–4.48)	
Post	0.59 (0.13–1.60)	0.60 (0.16–1.77)	0.49 (0.09–1.59)	0.61 (0.13–2.34)	
Percentage (%) change					
* Per protocol*	− 49.71 (− 81.98–3.50)	− 53.81 (− 84.73–9.09)	− 54.77 (− 82.76–29.22)	− 44.73 (− 81.22–14.09)	0.89
* As per treatment received*	− 51.56 (− 82.14–4.85)	− 54.84 (− 84.73–8.10)	− 55.41 (− 83.59–28.60)	− 62.59 (− 84.77–4.12)	0.86
**IL-6 (pg/mL)**					
Pre	4.95 (1.35–17.45)	5 (1.3–20.6)	6.15 (1.2–27.0)	4.9 (1.2–13)	
Post	2.7 (0.75–8.7)	2.8 (0.8–9.6)	2.25 (0.7–6.4)	3.3 (1.2–10.9)	
Percentage (%) change					
* Per protocol*	− 27.83 (− 78.91–98.21)	− 40.42 (− 79.86–71.42)	− 53.46 (− 84.29–23.21)	0 (− 66.79–142.46)	**0.007**
* As per treatment received*	− 27.83 (− 79.71–98.21)	− 40.42 (− 79.86–71.42)	− 51.54 (− 83.08–23.21)	− 16.66 (− 72–120)	0.08
**Trop I (ng/mL)**					
Pre	0.006 (0.001–0.007)	0.005 (0.001–0.01)	0.006 (0.001–0.01)	0.005 (0.001–0.007)	
Post	0.004 (0.001–0.006)	0.005 (0.001–0.008)	0.004 (0.001–0.006)	0.003 (0.001–0.006)	
Percentage (%) change					
* Per protocol*	− 11.98 (− 83.33–40)	0 (− 83.33–50)	− 33.33 (− 83.33–0)	0 (− 83.33–50)	0.55
* As per treatment received*	− 16.66 (− 83.33–40)	0 (− 83.33–40)	− 34.52 (− 83.33–0)	− 24.03 (− 83.33–50)	0.64

All values are Median (p25-p75); Bold P values are significant

*CRP* C-reactive protein, *IL-6* interleukin 6, *Trop I* troponin I, *IU* International Unit, *L* litre, *mg* milligram, *mL* millilitre, *pg* picogram, *ng* nanogram

Local lab ranges: CRP ≤ 0.06 mg/dl, IL6 = 0–4.4 pg/ml, Ferritin = 22–322 ng/ml, D-dimer ≤ 500 ng/ml, CPK = 32–294 IU/L and Troponin I ≤ 0.04 ng/ml

### Other outcomes

Of the 900 study participants, 25 (2.8%) patients had in-hospital mortality. The duration of hospital stay (P = 0.85), and the rates of clinical deterioration, i.e., ≥ 1 increase in baseline WHO Ordinal Scale (P = 0.68), requirement of mechanical ventilation (P = 0.48), progression to shock (P = 0.20), and in-hospital mortality (P = 0.46), were similar amongst the study groups (Table 3).

### Adverse events

In the aspirin arm (Group B), two patients discontinued the study drug (Additional file 1: eTable 1). One patient had two episodes of minor haemoptysis after three days of aspirin therapy. He had severe COVID-19 pneumonia which improved with conservative management. Another patient discontinued aspirin after two days of therapy due to petechiae. He had mild COVID that improved with conservative management. One patient in the atorvastatin arm (Group A) had severe myalgia after three days of therapy. It improved after the discontinuation of atorvastatin. This patient did not have raised serum CPK suggestive of myopathy or rhabdomyolysis. There were no statistically significant differences in adverse events across the study groups at the completion of the trial.

## Discussion

This open-label randomised controlled trial found no benefit of adding atorvastatin or aspirin or both to the standard of care in hospitalised patients with mild to moderate COVID-19. Additional treatment with aspirin, atorvastatin, or a combination of these did not prevent clinical deterioration to advanced disease in these patients when compared with standard of care. The other outcomes of in-hospital mortality, progression to shock, requirement of mechanical ventilation, and length of hospital stay were low and not altered by the study drugs. However, it was observed that aspirin and a combination of aspirin and atorvastatin reduced serum IL-6 levels during the hospital stay. Based on this trial, it cannot be commented on whether these drugs are useful in severe COVID-19 or not.

Enthusiasm for use of statins and aspirin as potential, repurposed, adjunctive drugs for the treatment of COVID-19 was sparked by in-vitro and in-vivo studies showing a reduction in inflammation and thrombosis in models of sepsis and pneumonias [2–5, 11, 12, 20, 21]. Statins have been demonstrated to inhibit the production of inflammatory cytokines in in-vitro studies [20] and redox markers in murine models of endotoxin-induced acute lung injury (ALI) [21]. Prior therapy with statins has been shown to be associated with reduced rates of severe sepsis and ICU admission in patients with acute bacterial and viral infections in observational studies [2–5].

Certain effects of statins might be especially beneficial in relation to coronaviruses. SARS-CoV-1 interacts with Toll-like receptors on the host cell membrane and significantly induces the expression of the MYD88 gene [22]. Downstream effects include activation of the NF-kB pathway and severe inflammation. Statins have been shown to stabilize MYD88 levels induced by a proinflammatory trigger in experimental models, thereby attenuating the inflammatory response [23]. A second theoretical anti-coronaviral action of statins involves interference with ACE2 signalling. SARS-CoV-2 utilizes ACE2 for initial entry and then down-regulates ACE2 expression. This action possibly activates the detrimental immune response and unopposed angiotensin II accumulation leading to organ injury [24]. Statins are known to up-regulate ACE2 via epigenetic modifications and thus may prove beneficial in COVID-19 infection [25].

Subsequent to these reports, a multitude of retrospective, observational studies reported on the use of statins in COVID-19. Meta-analyses of these studies suggested a reduction in fatal or severe disease with the use of statins [6–8]. However, other studies have shown equivocal results suggesting the need for a randomised control trial [9, 10].

Similarly, there has been an interest in repurposed use of aspirin for COVID-19 in view of reports of coagulopathy and life-threatening thrombotic events seen in severe COVID-19 infection [26, 27]. Similar to statins, pre-hospitalisation use of aspirin has been found to be strongly associated with decreased mortality in observational studies [15–17]. The postulated mechanisms include the antithrombotic properties and the anti-inflammatory action mediated through uncoupling of oxidative phosphorylation in hepatic mitochondria [28], induction of nitric oxide radicals [29] and modulation of NF-kB signalling [30].

In the past years, several clinical trials have tried to address the role of aspirin in COVID-19. Of these, the randomised trial conducted by the RECOVERY collaborative group has reported no reduction with aspirin in 28-day mortality or the risk of progression to invasive mechanical ventilation in patients hospitalised with COVID-19, irrespective of the severity of illness [18]. Aspirin was, however, associated with a small increase in the rate of being discharged from the hospital alive within 28 days. This multi-centre trial enrolled patients with relatively higher severity of illness and assessed all-cause mortality as the primary outcome. The median time of randomisation since symptom onset was 9 days (IQR 6–12 days).

The results of the observational studies of statins and aspirin in COVID-19 were not replicated in our clinical trial. One possible explanation for the discrepant findings could be inherent unknown confounders that bias observational studies and are eliminated in a randomised trial. Secondly, it is possible that the low event rates lead to a lack of power in the trial to detect the therapeutic efficacy of the intervention. The levels of inflammatory markers and troponin at baseline were lower than that reported in patients with severe COVID-19 indicating that the severity of the illness was mild to moderate. Thus, it is still possible that in cohorts with higher risk, these drugs may be useful as seen by the lowering of inflammatory marker IL-6 among patients on aspirin and the combination of aspirin and atorvastatin. However, this should be considered exploratory given the number of tests done. Another reason for the disparity could be that many of the observational trials showed benefit in patients with pre-hospitalisation use of aspirin and statin while we excluded these patients and added the drugs de-novo. Potentially, the effects of aspirin and statins could kick in early in preventing deterioration with prior use, while not being so useful once the effects of SARS-CoV-2 infection have already set in. However, this would be conjectural as the worst deterioration in COVID-19 happens during the cytokine storm which usually occurs a few days after the onset of symptoms.

Compared to the RECOVERY trial, our study examined the role of statins and aspirin in preventing clinical deterioration in patients with lesser severity of illness. The median time of randomisation since symptom onset was earlier at 6 days (IQR 4–8 days). The levels of inflammatory biomarkers were lower than that reported in patients with severe COVID-19. A relatively smaller number of patients were receiving LMWH and steroid at the time of randomisation. Patients with a WHO ordinal scale above 5 were excluded from our study. On the contrary, one-third of the patients in RECOVERY received respiratory support with NIV or invasive mechanical ventilation.

There were no significant adverse effects noted with the use of either of these drugs and they were safe to use in COVID-19 patients.

The strengths of our study include this being the first randomised controlled study to assess the efficacy of aspirin and statins in COVID-19, early recruitment at a median interval of 6 days (IQR 4–8 days) after the onset of symptoms, study of biochemical markers of inflammation and myocardial injury, and assessment with a clinically relevant ordinal scale.

The trial has several limitations. Some of these were related to logistic challenges like restricted access of research staff, limited access to day-to-day medical records, challenges in accessing past records of the patients, etc. This led to few ineligible patients getting randomised and a considerable number of participants not receiving the allocated study drug after randomisation. We tried to overcome this by doing ‘per-protocol’ and ‘as-treated’ analysis which revealed results similar to the modified ITT analysis. Another major limitation already discussed is the low event rate in the trial and lack of generalisability to patients with severe disease. Additionally, outcome ascertainment was limited to 10 days after randomization to facilitate the dissemination of findings. Also, the intent was to target the critical initial stages of inflammatory cascades (generally within 9–12 days after the onset of symptoms) that may initiate the cytokine storm. The short duration of treatment and follow-up precluded assessment of atherothrombotic risk associated with COVID that seems to persist for up to one year after infection.

## Conclusion

Among adults hospitalised with mild to moderate COVID-19 infection, the addition of aspirin, atorvastatin, or a combination of the two to the standard treatment did not prevent clinical deterioration. Aspirin and the combination of aspirin and atorvastatin did lead to reduction in serum IL-6 levels.

## Supplementary Information

**Additional file 1. eFigure 1.** Institute Covid-19 treatment protocol. **eFigure 2**. Probability of having WHO Ordinal Scale for Clinical Improvement < 6 in the study groups over time (Intension-to-treat analysis). **eFigure 3**. Probability of having WHO Ordinal Scale for Clinical Improvement < 6 in the study groups over time (Per protocol analysis). **eFigure 4**. Probability of having WHO Ordinal Scale for Clinical Improvement < 6 in the study groups over time (as treated analysis). **eTable 1**. Distribution of adverse events in the study groups.

## Data Availability

The datasets generated during and analyzed during the current study are not publicly available as the data is not in a public repository. We would not like to share the data pending provisional acceptance of the article. But deidentified participant data, study protocol, statistical analysis plan, informed consent forms will be made available with publication from the corresponding author on reasonable request.
