# Supplementary material for: Statin and aspirin as adjuvant therapy in hospitalised patients with SARS-CoV-2 infection: a randomised clinical trial (RESIST trial)
Source: BMC Infect Dis. 2022 Jul 9;22:606. doi: 10.1186/s12879-022-07570-5 (PMC9270743; doi:10.1186/s12879-022-07570-5)
Supplement: Supplementary file 1 — Additional file 1. eFigure 1. Institute Covid-19 treatment protocol. eFigure 2. Probability of having WHO Ordinal Scale for Clinical Improvement < 6 in the study groups over time (Intension-to-treat analysis). eFigure 3. Probability of having WHO Ordinal Scale for Clinical Improvement < 6 in the study groups over time (Per protocol analysis). eFigure 4. Probability of having WHO Ordinal Scale for Clinical Improvement < 6 in the study groups over time (as treated analysis). eTable 1. Distribution of adverse events in the study groups. [file 12879_2022_7570_MOESM1_ESM.docx]

**Statin and Aspirin as Adjuvant Therapy in Hospitalised Patients with SARS-CoV-2 Infection: A Randomized Clinical Trial (RESIST Trial)**

**Supplementary Appendix**

| **Contents** | **Page number** |
| --- | --- |
| **eFigure 1**: Institute Covid-19 treatment protocol | **2** |
| **eFigure 2:** Probability of having WHO Ordinal Scale < 6 in the study groups over time  (True ITT analysis) | **3** |
| **eFigure 3**: Probability of having WHO Ordinal Scale < 6 in the study groups over time (Per protocol analysis) | **4** |
| **eFigure 4**: Probability of having WHO Ordinal Scale for Clinical Improvement < 6 in the study groups over time (As treated analysis) | **5** |
| **eTable 1:** Distribution of adverse events in the study groups | **6** |

**
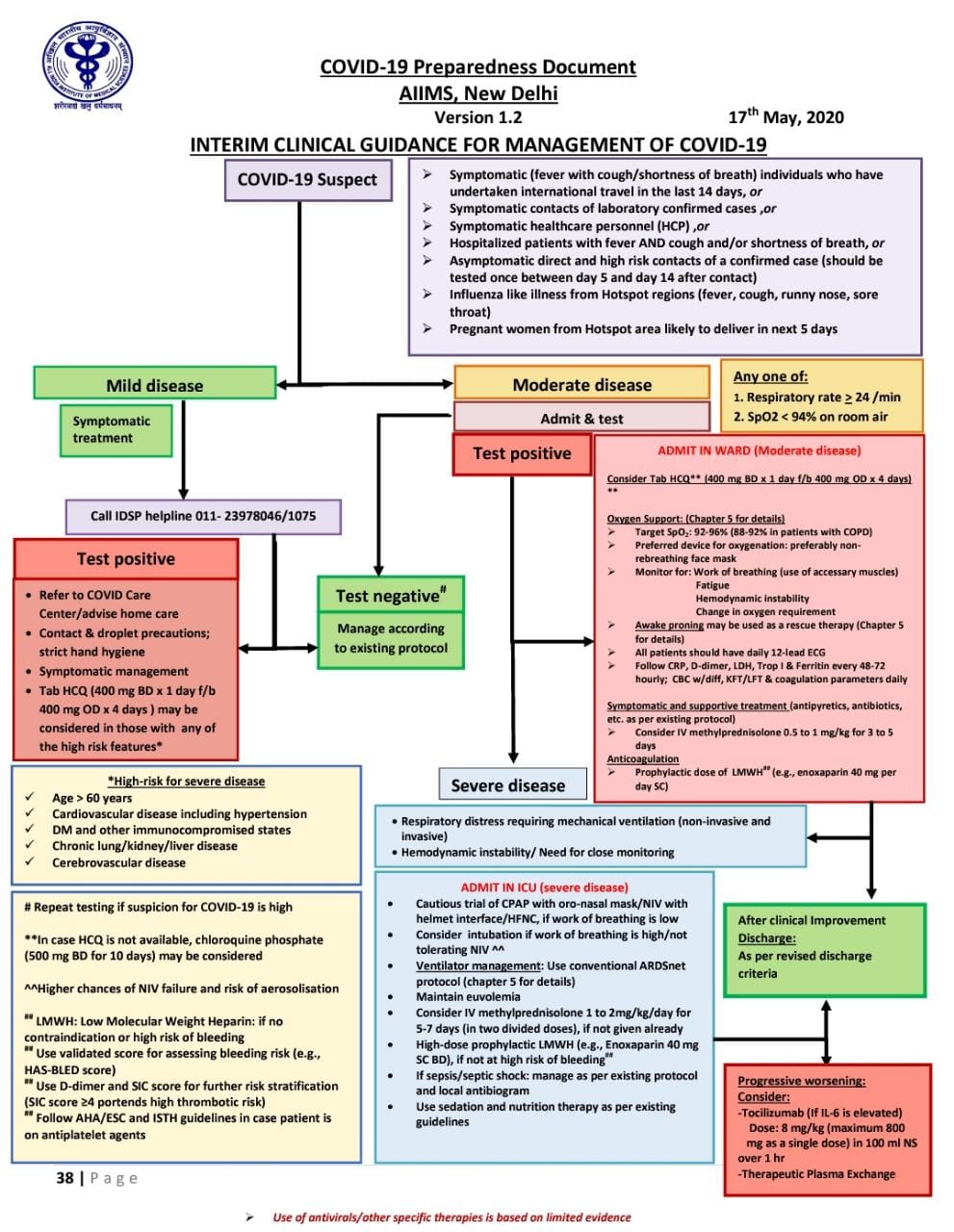
**

**eFigure 1:** Institute Covid-19 treatment protocol

**
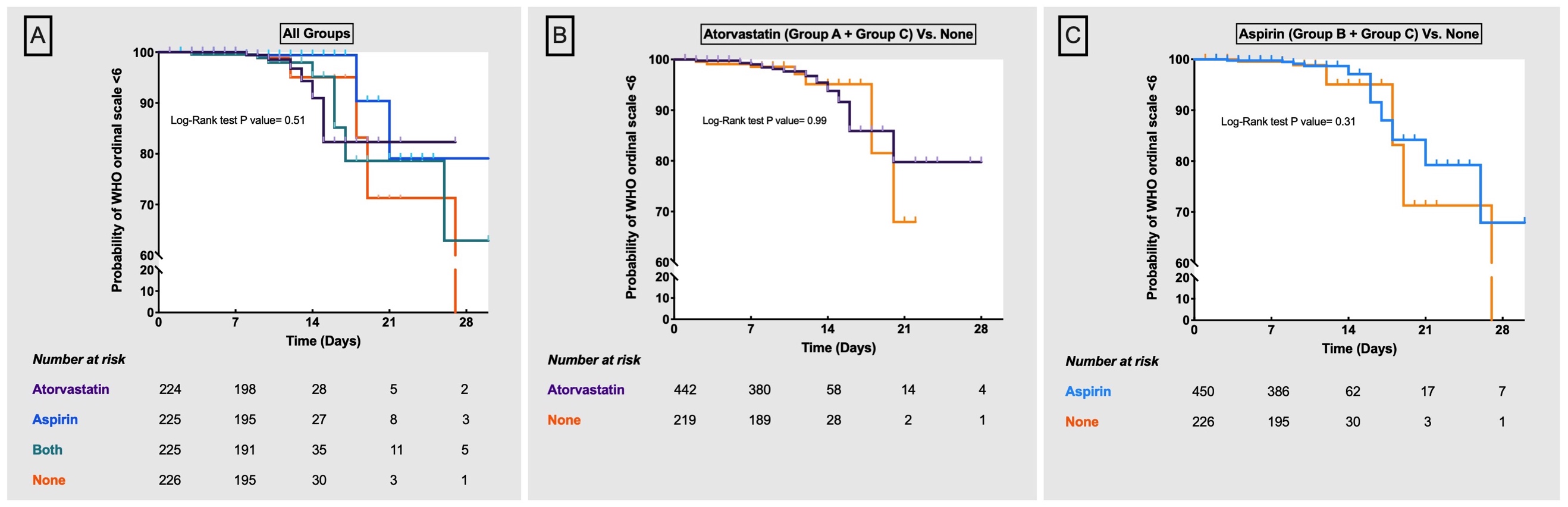
eFigure 2:** Probability of having WHO Ordinal Scale for Clinical Improvement < 6 in the study groups over time (Intension-to-treat analysis)

***Panel A*** *is showing Kaplan-Meier estimates of the freedom from primary outcome after initiation of the study drugs (atorvastatin, aspirin, and both) in comparison to the standard of care (ITT analysis).* ***Panel B and C*** *are showing Kaplan-Meier estimates of probability of freedom from primary outcome in combined atorvastatin (Group A and Group C) and combined aspirin (Group B and Group C) groups respectively in comparison to the standard of care (ITT analysis). CI= Confidence Interval, HR= Hazard ratio, WHO= World health organisation*


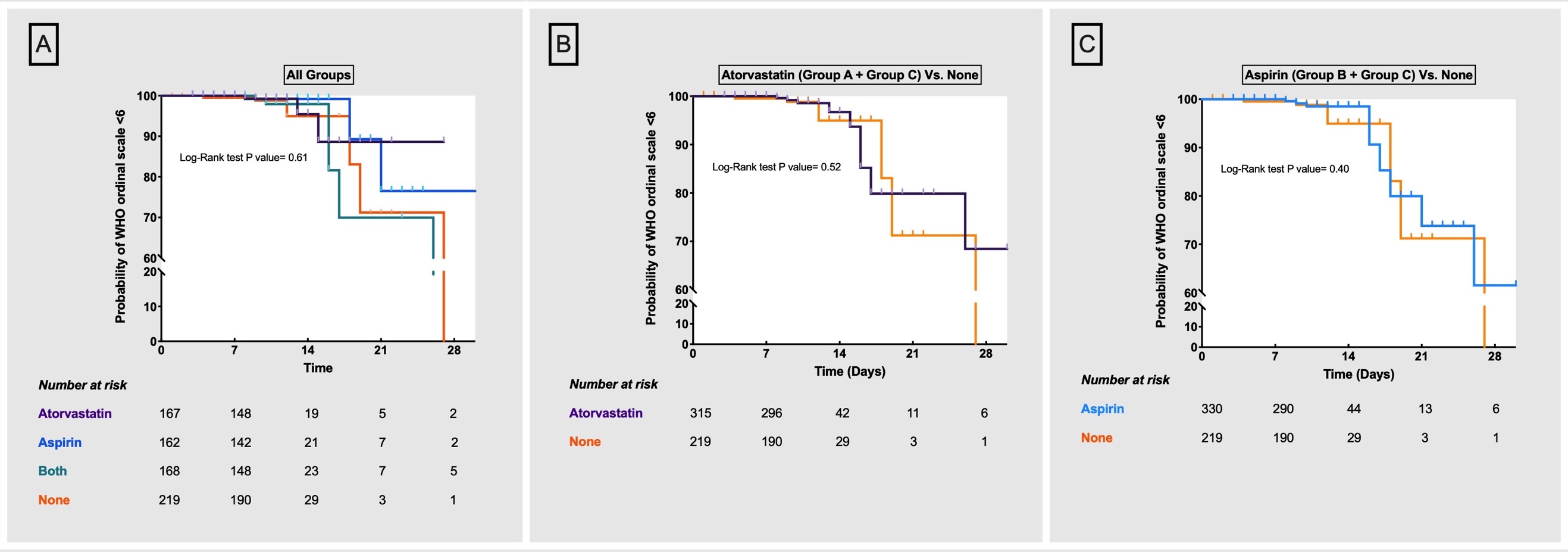


**eFigure 3:** Probability of having WHO Ordinal Scale for Clinical Improvement < 6 in the study groups over time (Per protocol analysis)

***Panel A*** *is showing Kaplan-Meier estimates of the freedom from primary outcome after initiation of the study drugs (atorvastatin, aspirin, and both) in comparison to the standard of care (Per protocol analysis).* ***Panel B*** ***and C*** *are showing Kaplan-Meier estimates of probability of freedom from primary outcome in combined atorvastatin (Group A and Group C) and combined aspirin (Group B and Group C) groups respectively in comparison to the standard of care (Per protocol analysis). CI= Confidence Interval, HR= Hazard ratio, WHO= World health organisation*


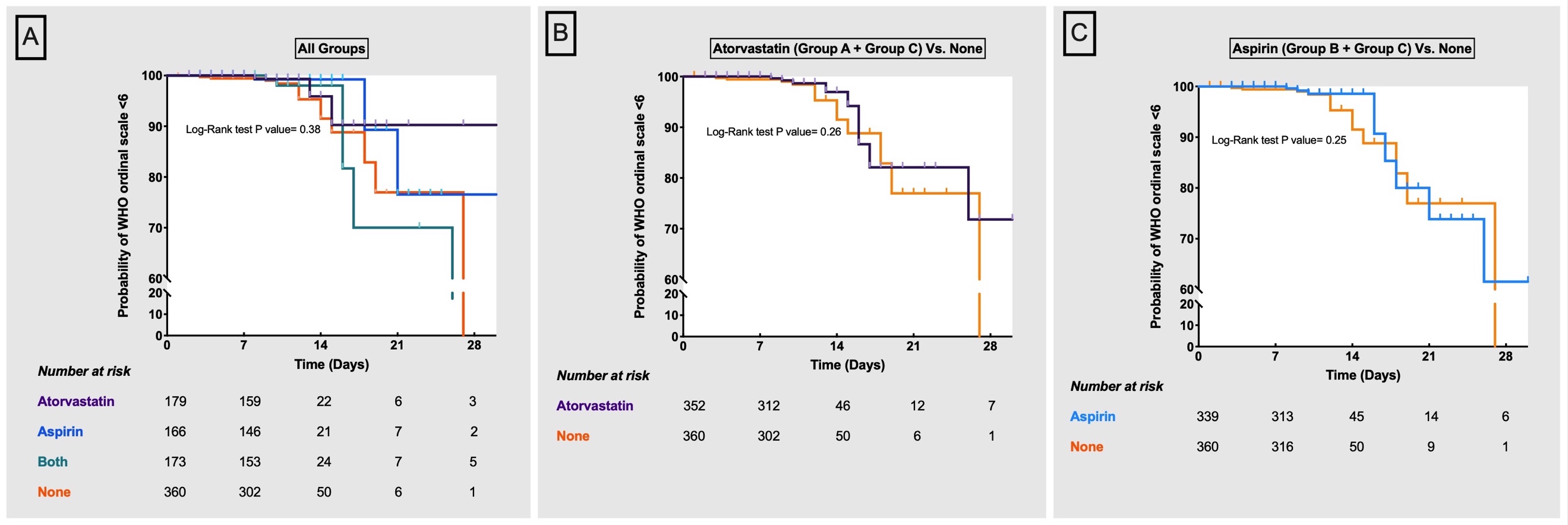


**eFigure 4:** Probability of having WHO Ordinal Scale for Clinical Improvement < 6 in the study groups over time (as treated analysis)

***Panel A*** *is showing Kaplan-Meier estimates of the freedom from primary outcome after initiation of the study drugs (atorvastatin, aspirin, and both) in comparison to the standard of care (As treated analysis).* ***Panel B and C*** *are showing Kaplan-Meier estimates of probability of freedom from primary outcome in combined atorvastatin (Group A and Group C) and combined aspirin (Group B and Group C) groups respectively in comparison to the standard of care (As treated analysis). CI= Confidence Interval, HR= Hazard ratio, WHO= World health organisation*

**eTable 1**: Distribution of adverse events in the study groups

| **Adverse events** | **Group A**  **(Atorvastatin)** | **Group B**  **(Aspirin)** | **Group C**  **(Both)** | **Group D**  **(None)** | **P Value** |
| --- | --- | --- | --- | --- | --- |
| **Myalgia ^a^** |  |  |  |  |  |
| Modified Intention to treat | 1/221 (0·4) | 0/221 (0) | 0/221 (0) | 0/219 (0) | - |
| Per protocol | 1/167 (0·6) | 0/162 (0) | 0/168 (0) | 0/219 (0) | - |
| As per treatment received | 1/179 (0·6) | 0/166 (0) | 0/173 (0) | 0/360 (0) | - |
| **Myopathy ^b^** |  |  |  |  |  |
| Modified Intention to treat | 0/221 (0) | 0/221 (0) | 0/221 (0) | 0/219 (0) | - |
| Per protocol | 0/167 (0) | 0/162 (0) | 0/168 (0) | 0/219 (0) | - |
| As per treatment received | 0/179 (0) | 0/166 (0) | 0/173 (0) | 0/360 (0) | - |
| **Rhabdomyolysis ^c^** |  |  |  |  |  |
| Modified Intention to treat | 0/221 (0) | 0/221 (0) | 0/221 (0) | 0/219 (0) | - |
| Per protocol | 0/167 (0) | 0/162 (0) | 0/168 (0) | 0/219 (0) | - |
| As per treatment received | 0/179 (0) | 0/166 (0) | 0/173 (0) | 0/360 (0) | - |
| **Raised Liver enzymes ^d^** |  |  |  |  |  |
| Modified Intention to treat | 6/221 (2·7) | 11/221 (4·9) | 10/221 (4·5) | 13/219 (5·9) | 0.426 |
| Per protocol | 6/167 (3·6) | 11/162 (6·8) | 10/168 (5·9) | 13/219 (5·9) | 0.616 |
| As per treatment received | 8/179 (4·5) | 11/166 (6·6) | 10/173 (5·8) | 21/360 (5·8) | 0.853 |
| **Minor bleeding ^e^** |  |  |  |  |  |
| Modified Intention to treat | 0/221 (0) | 2/221 (0·9) | 0/221 (0) | 0/221 (0) | - |
| Per protocol | 0/167 (0) | 2/162 (1·2) | 0/168 (0) | 0/219 (0) | - |
| As per treatment received | 0/179 (0) | 2/166 (1·2) | 0/173 (0) | 0/360 (0) | - |
| **Major bleeding ^f^** |  |  |  |  |  |
| Modified Intention to treat | 0/221 (0) | 0/221 (0) | 0/221 (0) | 0/219 (0) | - |
| Per protocol | 0/167 (0) | 0/162 (0) | 0/168 (0) | 0/219 (0) | - |
| As per treatment received | 0/179 (0) | 0/166 (0) | 0/173 (0) | 0/360 (0) | - |

^a^ Severe muscle pain or aches (CPK < ULN)

**^b^** Unexplained muscle pain or weakness accompanied by CPK >10 x ULN

**^c^** Severe myopathy with CPK >40 x ULN and myoglobinuria ± acute renal failure)

**^d^** ALT/AST > 3 x ULN

**^e^** BARC bleeding type 1 and 2 i.e., bleeding that is not actionable and does not cause the patient to seek treatment, bleeding requiring a healthcare assessment or less invasive treatment such as heavy menstrual bleeding, ecchymosis, or epistaxis etc.

**^f^** BARC bleeding type ≥ 3 i.e., significant blood loss requiring blood transfusion, bleeding into a critical closed space (e.g., intracranial bleeding, compartment syndrome), bleeding requiring an intervention for management (e.g., surgery, interventional radiology procedures, endoscopic treatments), and fatal bleeding.
